# Hospital Committees, Trained Nurses, and the Public

**Published:** 1892-07-16

**Authors:** 


### The Hospital

An Institutional Journal of

Science, Medicine, Nursing, and Philanthropy.

*For Hospitals, Asylums, Infirmaries, Dispensaries, Medical Practitioners, Students, Nurses, and the Charitable Public.*

Vol. XII.—No. 303.] [July 16, 1892.

### Hospital Committees, Trained Nurses and the Public.

Now that sufficient time has elapsed to enable the report of the Lords' Committee to be carefully studied, it may not be inappropiate to consider the relations that should subsist between Hospital Committees, Trained Nurses, and the Public. It is only during the last ten years that there have been trained nurses in any true acceptation of the term. At the present time no nurse is entitled to be considered trained who has not (1) resided for a period of at least twelve months in a hospital containing 100 beds, where she has attended lectures and received technical training of an adequate and practical character; and (2) for two subsequent years been engaged in learning other branches of nursing not easily, if at all, to be acquired in a hospital. The latter two years should be devoted—six months to acquiring the art of private nursing by attending patients at their own homes; six months to the study of midwifery, massage, and women's cases generally; six months to nursing in a fever hospital; and six months to district nursing. Any woman who thus apportions her time will, with ordinary intelligence, at the expiration of three years be entitled to consider herself a trained nurse. The fact that such a training as we have roughly indicated should be necessary, will make it easy for those, who have no technical knowledge of the subject, to understand that at the present time there are relatively few trained nurses in the country, although the number is largely increasing every year owing to the improved system of training which is now obtainable. Trained nursing may therefore be said to be at the present time in its initial stage, and it is most important that no retrograde step should be taken by the setting up of a system such as registration, which the Loids' Committee justly show will not secure the objects which its advocates desire. Everything should be done out of fullness of knowledge, and with the set purpose of securing for nursing as a calling the high position it ought to occupy in the estimation of the world.

In these circumstances it is opportune to consider the position which Hospital Committees occupy in relation to (1) Trained Nurses; and (2) the General Public.

The general public are dependent upon the Hospital Committees for the instruction, training, and supply of nurses at the present time. The demand for skilled nurses has forced Hospital Committees to establish institutions in connection with their hospitals from which nurses may be obtained by the general public. These institutions are not only self-supporting but often bring considerable additions to the funds of the hospitals. In these circumstances the Hospital Committee's duty is clear and precise. Every trained nurse working for an institution connected with a hospital should be provided with a separate sleeping-room, with full uniform, a comfortable home when she is not at a case, adequate remuneration based upon a sliding scale commission on her earnings, and sick pay and pension when incapacitated for work. It is clearly the duty of the members of the committees and medical staffs, and also of the governors at large to see that every trained nurse attached to the hospital with which they are connected shall be properly housed, adequately cared for, and that her remuneration shall be based upon the co operative principle, seeing, as the Lords' Committee point out, the large amount of money a nurse can earn for her hospital. Further than this, as the Lords' Committee urge, it is very desirable that pensions should be provided for nurses, either "by the hospital, following the example of the London and Guy's, by joining the National Pension Fund for Nurses, or by the hospital providing a special pension out of its own funds." Of course, each Hospital Committee will have to adopt the course which is the least costly to the institution, and for this reason they will no doubt ultimately elect to become affiliated to the Royal National Pension Fund, as that will entail far less outlay, and will confer far greater advantages on their nursing staff than any other method.

The duties of Hospital Committees to the general public may be briefly stated. They are to give the public the necessary information to enable anyone to secure the services of a trained nurse of the highest character, whenever such services may be required; to protect private patients from improper and incapable women calling themselves nurses, who have in the past found their way into private houses, to the discomfort of the inmates and to the serious injury of skilled nursing; and to open out for grateful patients a ready means of showing their gratitude to nurses who may have attended them during serious illness by affording them an opportunity of contributing a sum to the Pension Fund the whole of which shall be invested for the nurse's benefit. This last point is one of real importance, because it would put a stop to the waste due to the purchase of presents for the nurses in such circumstances, which are often of no real value to the receiver, although they may have cost considerable sums of money.

Having thus dealt with the position which Hospital Committees should occupy in relation to trained nurses and the general public, we pass on to consider the three interests in question as a whole. What would constitute an ideal system so far as hospital authorities, trained nurses, and the public are concerned? Clearly one which would secure that each hospital of standing should provide an adequate number of trained nurses, who should receive such remuneration and privileges as would make them independent and happy, and at the same time afford the public a reasonable guarantee that they might engage the services of a nurse with the assured certainty that those services would prove of the highest value. We have at the present time an increasing nnmber of hospitals with one hundred beds and upwards, which lay themselves out to train nurses on an intelligent system, and to supply them to the public through the private institution which they have established. In some cases the hospital authorities have agreed to pay the nurses on the private staff a sliding scale commission on their earnings as a fair addition to their regular hospital wages, and also to commence tentatively a plan for providing pensions and sick pay.

One result of the Lords' Committee should be to secure that every hospital which trains and supplies nurses shall at once proceed to consider the regulations governing this branch of their work with a view to introducing such amendments as may be necessary to bring the whole system up to the highest standard of perfection in the interests of everybody concerned. Strenuous efforts should be made to teach the public that in order to secure a trained nurse of reliability and character, it is safest and best to communicate at once with the matron of the hospital to which they subscribe, or with which they are familiar, and to ask her to send a nurse suitable for the particular case, the nature of which should be certified by the doctor in attendance. In case the nurse should not give satisfaction, the employer should be encouraged to communicate with the authorities of the institution from which she was received so that an immediate investigation may be made, and justice may be done to nurse and patient alike. In this way an immense public service would be rendered by the hospitals to the country at large, whilst substantial equity would be secured for the nurses whose interests we have very warmly at heart.

One portion of the report of the Lords' Committee, that which deals with registration, has convinced the public and impartial people generally, that whether the attempt to enforce it be persisted in or not, a register of trained nurses under the conditions laid down by the advocates of nurse registration would be no protection to the public, and anything but an advantage to the nurses. In these circumstances the registration of nurses may for practical purposes be considered of no further interest, whether the promoters of the scheme determine to persist in it, or to show, by admitting the inutility and impracticability of their proposals, that they really have at heart the advancement of good nursing and the welfare of trained nurses. In any case the responsibility of protecting the public is now thrown upon the hospital authorities and nurse-training schools. It is for them, or for the majority of them, to show that they desire to help the public by giving them a guide which shall effectually enable anyone unacquainted with nursing to secure, in case of illness, the skilled services which they need.

How is this to be done? Every nurse-training school has a list or [register of nurses who have been trained and certificated at such school. At the present time a committee of representatives of the nurse-training schools is sitting, and if, as we understand to be the case, the more intelligent authorities are prepared to co-operate, there need be no delay in securing great popularity by helping the public to help themselves. All that is necessary is to appoint a representative committee with power to prepare and publish an "Official Directory of Trained Nurses," under the authority of the nurse-training schools. This committee would edit the "Official Directory of Trained Nurses," and it would be their duty to see that the name of every nurse who had been adequately trained, whether she is certificated or not, was published in the "Directory," and that such name appeared there year after year unless, or until, the nurse did something which in the opinion of the Editorial Committee, on the representation of the authorities of the school where she was trained, rendered it desirable or necessary to omit her name from such a book. The particulars contained in the "Directory" would simply be the full name of the nurse, details of her training, and subsequent experience, and her special qualifications, if any. Anyone who engaged a nurse whose name appeared in the Official Directory, if such nurse proved inefficient, could make a complaint to the Matron of the school where the nurse was trained, the authorities of which would first go into the matter, having at heart the best interests of the nurse, which would also be those of the institution. Such charges would then be dealt with on their merits, and in the graver cases it would be within the discretion of the authorities of the training school to communicate with the Editorial Committee of the Official Directory as a sort of Court of Appeal, so that everything would be done with the utmost deliberation and care, and all the interests concerned would thus be adequately protected. If it be true that the suggestion of an Official Directory, though frowned upon by some authorities, is favoured by others, we are of opnion that it is of the first importance to the well-being of trained nurses and to the best interests of the nurse-training schools that such an Official Directory should be published under authority without any unnecessary delay. Hospitals and nurse-training schools must move with the times, and now that they have shown so convincingly that the registration of nurses will be no protection to the public, whilst it will tend to reduce nurses to one common level, it is not too much to urge them to display a little public spirit by combining to give the public protection, and all trained nurses a proper standing and position by the publication of the Official Directory, which we have not failed to advocate on several previous occasions.

